# The experience of the Kiev regional AIDS center in combating the spread of HIV infection in the Kiev and interaction with non-governmental social organization

**DOI:** 10.1186/1471-2334-14-S2-P25

**Published:** 2014-05-23

**Authors:** Oleksandr Yurchenko, Tetiana Stepchenkova

**Affiliations:** 1 Kiev regional AIDS center, Kyiv, Ukraine

## 

Since the beginning of the registration of HIV, in Kiev there were registered a total 13,500 cases of HIV, 1,5 thousands people died. Under medical supervision in Kiev there are 9697 people with HIV, including the 1790 people who are in the stage of AIDS. Among vulnerable groups particularly distinguished IDU. During 2013 the number tested for HIV among this group is 3900 people. Infection level among them is 19.5 %. Identified by year of HIV-positive injecting drug users - 752. Enrollment rate in 2013 is 64 %. The majority (70.6 %) from infected IDU’s have III –IV clinical stage of disease. The assistance of non-governmental social organizations is critical. With the number of employees in the region of NGOs involved in the implementation of measures to combat HIV / AIDS in the region, the 5 most productive organizations are supported by the administration of Kyiv regional Clinical Hospital № 5 by allocating space for activities. Based cabinets anonymous HIV testing within the network Kyiv City AIDS Center and sites of ART also established cooperation with AIDS-service NGOs that implement preventive work with vulnerable groups and provide social support for persons living with HIV. Social workers allocated by the rooms required for their activity. Other NGOs engaged in addressing transition to the offices of anonymous testing people, who were positive in a testing in community centers, the mobile clinic or while.

Results cooperation center and HIV service NGOs in Kiev:

• Of the 932 patients, who were appointed ART, 894 patients (95.9 %) under the guidance of social workers

• Percentage of adults and children with HIV known to be on ART at 12 months after initiation of ART- 86.43 %

• Percentage of HIV -infected persons who received outpatient examination at the end of the year among the total number of HIV-infected persons who are under medical supervision 82%

• Since 2009 increased the percentage of HIV -infected with TB who received treatment for TB and HIV, including through implementation of measures of social support persons with a triple problem of injecting drug users, HIV, TB

